# Supplementary material for: The research data management platform (RDMP): A novel, process driven, open-source tool for the management of longitudinal cohorts of clinical data
Source: Gigascience. 2018 May 22;7(7):giy060. doi: 10.1093/gigascience/giy060 (PMC6041881; doi:10.1093/gigascience/giy060)
Supplement: GIGA-D-18-00070_Revision_1.pdf [file giy060_giga-d-18-00070_revision_1.pdf]

GigaScience

The Research Data Management Platform (RDMP): A novel, process driven, open-source tool for the management of longitudinal cohorts of clinical data

--Manuscript Draft--

Manuscript Number:	GIGA-D-18-00070R1
Full Title:	The Research Data Management Platform (RDMP): A novel, process driven, open-source tool for the management of longitudinal cohorts of clinical data
Article Type:	Technical Note
Funding Information:	Medical Research Council (MR/M501633/1) — Not applicable<br>Wellcome Trust (WT086113) — Not applicable<br>Horizon 2020 (633983) — Not applicable
Abstract:	Background: The Health Informatics Centre (HIC) at the University of Dundee provides a service to securely host clinical datasets and extract relevant data for anonymised cohorts to researchers to enable them to answer key research questions. As is common in research using routine healthcare data, the service was historically delivered using ad-hoc processes resulting in the slow provision of data whose provenance was often hidden to the researchers using it. This paper describes the development and evaluation of the Research Data Management Platform (RDMP): an open source tool to load, manage, clean, and curate longitudinal healthcare data for research and provide reproducible and updateable datasets for defined cohorts to researchers.<br><br>Results: Between 2013 and 2017, RDMP tool implementation tripled the productivity of Data Analysts producing data releases for researchers from 7.1 to 25.3 per month; and reduced the error rate from 12.7% to 3.1%. The effort on data management reduced from a mean of 24.6 to 3.0 hours per data release. The waiting time for researchers to receive data after agreeing a specification reduced from approximately 6 months to less than one week. The software is scalable and currently manages 163 datasets. 1,321 data extracts for research have been produced with the largest extract linking data from 70 different datasets.<br><br>Conclusions: The tools and processes that encompass the RDMP not only fulfil the research data management requirements of researchers but also support the seamless collaboration of data cleaning, data transformation, data summarisation and data quality assessment activities by different research groups.
Corresponding Author:	Emily Jefferson<br><br>UNITED KINGDOM
Corresponding Author Secondary Information:	
Corresponding Author's Institution:	
Corresponding Author's Secondary Institution:	
First Author:	Thomas Nind
First Author Secondary Information:	
Order of Authors:	Thomas Nind
	James Galloway
	Gordon McAllister
	Donald Scobbie

Powered by Editorial Manager® and ProduXion Manager® from Aries Systems Corporation

	Wilfred Bonney
	Christopher Hall
	Leandro Tramma
	Parminder Reel
	Martin Groves
	Philip Appleby
	Alex Doney
	Bruce Guthrie
	Emily Jefferson
**Order of Authors Secondary Information:**	
**Response to Reviewers:**	Dear Dr Edmunds<br><br>Thank you for your email dated 4th May 2018 enclosing the reviewers' comments. We have carefully reviewed the comments and have revised the manuscript accordingly. Our responses are given in a point-by-point manner in the text below.<br><br>We hope the revised version is now suitable for publication and look forward to hearing from you in due course.<br><br>Sincerely<br>Emily Jefferson, PhD<br><br><br><br>1st Reviewer: No Changes suggested<br><br>2nd Reviewer<br><br>1) Length of background:<br>Reviewer comments: The background is quite long, and it takes a while to get to the "what". The problem should be well known to the journal audience, I don't need to be convinced that this is a significant challenge with importance to the larger research community. I would suggest getting to the description/solution (and figure 2) sooner.<br><br>Changes on p.3-4:<br>We have removed some superfluous words throughout the Background section.<br><br>We removed from the first paragraph:<br>"In the UK, there are a number of Research Data Management (RDMP) programmes sponsored by the Joint Information Systems Committee (JISC), a government-funded body charged with driving forward innovation and the use of digital technologies in UK higher education and research [6]. Although established in the humanities (Sudamih [7, 8]) and life sciences (ADMIRAL, [9]),"<br><br>We have removed from the section describing why the existing RDM tools and off-the-shelf tools did not meet HIC's requirements the following text:<br>•Data Cleaning and Curation Tools: "A considerable effort would be needed to develop a data cleaning and processing pipeline before being able to use many of the existing RDM tools."<br>•Complex extraction transforms: "If there are 10 optional transforms on a dataset which can be mixed and matched, then a view based approach does not scale to such a solution."<br>•Data Lifecycle Management: "during the requirements gathering stage of this project, the opinions of how to clean and standardise data were sought from many different researchers and clinicians who had previously been provided with research extracts for analysis by HIC. It was".<br><br>From the paragraph describing the Repository Data Lifecycle and Project Data

Powered by Editorial Manager® and ProduXion Manager® from Aries Systems Corporation

	Lifecycle we have removed: “Figure 1 summarises the integrated data management lifecycle used by the RDMP.” and “Not all projects have to use the same transforms.”<br><br>We have reduced the total length of the Background section by 17%.<br><br>2) How is privacy handled:<br>Reviewer Comments: I think it would be useful to add a section describing how privacy is handled. Will the ability to export a dataset without PII, a certifiable de-identified form or other option be added? Right now this seems to be the researcher's responsibility but it could be helpful to bake into the workflow and/or automate some elements of different jurisdictions etc. For example, could you recognize id's (SSN etc.), addresses, postcodes, phone numbers, email addresses and confirm they are meant to be included. Specify these elements and allow two forms of export (one de-identified and one identified), understandably this may be more suitable for a future release.<br><br>Changes on p.7.<br>We have added the following section:<br><br>“Privacy Handling<br>Ensuring identifiable data does not appear in data extractions is principally done by configuring which columns are extractable, which require special governance approval to be extracted and which contain patient identifiers (and therefore should not be extracted). This can be done once per dataset after which the rules will be applied to all project extractions. Since manual processes can be error prone the release pipeline also be adjusted to include further blanket checks. For example, adding the 'ColumnBlacklister' component to the default extraction pipeline allows specification of a Regular Expression which will block any data extractions containing columns matching the pattern (e.g. containing the word 'Id', 'Address' or 'Identifier'). It is also possible to write custom plugin data flow components. One such plugin component used by HIC looks for 10 digit sequences where the checksum matches the Scottish patient identifier CHI (Community Health Index) checksum algorithm in data being extracted. This prevents CHI numbers appearing in free text/unexpected fields from being extracted.”<br><br>Recommended changes by Editor:<br>Please register any new software application in the SciCrunch.org database to receive a RRID (Research Resource Identification Initiative ID) number, and include this in your manuscript.<br><br>Changes made on page 15:<br>We have added under the Availability of source code and requirements section:<br>“SciCrunch RRID (Research Resource Identification Initiative ID): Research Data Management Platform, RRID:SCR_016268”<br><br>The instructions to authors states:<br>“GigaScience requires authors to deposit the data set(s) supporting the results reported in submitted manuscripts in a publicly-accessible data repository, such as GigaDB (see GigaDB database terms of use for complete details). This section should be included when supporting data are available and must include the name of the repository and the permanent identifier or accession number and persistent hyperlinks for the data sets (if appropriate)”<br><br>Changes made on page 15:<br>As per submission requirements we have uploaded the data used for the analysis of the paper<br>“The datasets supporting the results of this article are presented in a file named Data and Analysis.rar are available via the GigaScience repository: "The Research Data Management Platform (RDMP)" GigaScience Database.<br>http://dx.doi.org/10.5524/100455.”
**Additional Information:**	
**Question**	**Response**

Are you submitting this manuscript to a special series or article collection?	No
**Experimental design and statistics**<br><br>Full details of the experimental design and statistical methods used should be given in the Methods section, as detailed in our Minimum Standards Reporting Checklist. Information essential to interpreting the data presented should be made available in the figure legends.<br><br>Have you included all the information requested in your manuscript?	Yes
**Resources**<br><br>A description of all resources used, including antibodies, cell lines, animals and software tools, with enough information to allow them to be uniquely identified, should be included in the Methods section. Authors are strongly encouraged to cite Research Resource Identifiers (RRIDs) for antibodies, model organisms and tools, where possible.<br><br>Have you included the information requested as detailed in our Minimum Standards Reporting Checklist?	Yes
**Availability of data and materials**<br><br>All datasets and code on which the conclusions of the paper rely must be either included in your submission or deposited in publicly available repositories (where available and ethically appropriate), referencing such data using a unique identifier in the references and in the “Availability of Data and Materials” section of your manuscript.<br><br>Have you have met the above requirement as detailed in our Minimum Standards Reporting Checklist?	Yes

Manuscript

Click here to download Manuscript The Research Data Management Platform (RDMP)_SCE_ERJ.docx

Title Page

The Research Data Management Platform (RDMP): A novel, process driven, open-source tool for the management of longitudinal cohorts of clinical data

Authors' Information

Thomas Nind[1] (t.z.nind@dundee.ac.uk)

James Galloway[1] (j.r.galloway@dundee.ac.uk)

Gordon McAllister[1] (gordonmc8@gmail.com)

Donald Scobbie[2] (d.scobbie@epcc.ed.ac.uk)

Wilfred Bonney[1] (wbonney@dal.ca)

Christopher Hall[1] (c.hall@dundee.ac.uk)

Leandro Tramma[1] (l.tramma@dundee.ac.uk)

Parminder Reel[1] (p.s.reel@dundee.ac.uk)

Martin Groves[1] (m.groves@dundee.ac.uk)

Philip Appleby[1] (p.appleby@dundee.ac.uk)

Alex Doney[1] (A.Doney@dundee.ac.uk)

Bruce Guthrie[1] (B.Guthrie@dundee.ac.uk, ORCID: 0000-0003-4191-4880)

Emily Jefferson[1]* (e.r.jefferson@dundee.ac.uk, ORCID: 0000-0003-2992-7582)

[1]Health Informatics Centre, University of Dundee, Mail Box 15, Ninewells Hospital & Medical School, Dundee, DD1 9SY, UK

[2]Edinburgh Parallel Computing Centre, James Clerk Maxwell Building, Peter Guthrie Tait Road, Edinburgh, EH9 3FD, UK

*Corresponding Author

Abstract

Background: The Health Informatics Centre (HIC) at the University of Dundee provides a service to securely host clinical datasets and extract relevant data for anonymised cohorts to researchers to enable them to answer key research questions. As is common in research using routine healthcare data, the service was historically delivered using ad-hoc processes resulting in the slow provision of data whose provenance was often hidden to the researchers using it. This paper describes the development and evaluation of the Research Data Management Platform (RDMP): an open source tool to load, manage, clean, and curate longitudinal healthcare data for research and provide reproducible and updateable datasets for defined cohorts to researchers.

Results: Between 2013 and 2017, RDMP tool implementation tripled the productivity of Data Analysts producing data releases for researchers from 7.1 to 25.3 per month; and reduced the error rate from 12.7% to 3.1%. The effort on data management reduced from a mean of 24.6 to 3.0 hours per data release. The waiting time for researchers to receive data after agreeing a specification reduced from approximately 6 months to less than one week. The software is scalable and currently manages 163 datasets. 1,321 data extracts for research have been produced with the largest extract linking data from 70 different datasets.

Conclusions: The tools and processes that encompass the RDMP not only fulfil the research data management requirements of researchers but also support the seamless collaboration of data cleaning, data transformation, data summarisation and data quality assessment activities by different research groups.

Keywords: Clinical Datasets, Translational Research, Research Data Management, Data Catalogue, Health Informatics, Record Linkage

Background

In recent years, many academic institutions have taken significant roles in the management of research data by promoting a research data lifecycle as a concept to support data acquisition, curation, preservation, sharing, and reuse of healthcare data [1-4]. Pilot RDM programmes in biomedicine (MaDAM and MiSS [5]) have been established including tools such as i2b2 [6], STRIDE [7], CSDMSs [8], ClinData Express [9], REDCap [10] and tranSMART [11, 12]. These tools are often used by research institutions to manage consented longitudinal cohorts of data.

Healthcare systems also regularly provide data for research alongside their primary role of managing data for administrative purposes (e.g. National Health Service (NHS) Digital in England or NHS Information Services Division (ISD) in Scotland). Such organisations tend to provide data extracts for specific cohorts as one-off extracts rather than manage longitudinal cohorts and use generic industry standard IT tools such as Business Objects for data management and extraction.

In the Tayside and Fife regions of Scotland, there is a long history of close partnership between the University of Dundee and local NHS Health Boards who are responsible for delivering healthcare to all residents in a geographical region. This has allowed a continuous feed of longitudinal clinical and research datasets to the Health Informatics Centre (HIC), with some datasets now containing over 50 years of historical data [13]. HIC provides a service to securely host 163 datasets and to extract relevant linked anonymised data for researcher use to enable them to answer key research questions.

Prior to 2014, HIC used Microsoft's SQL Server Integration Services for loading data and then hand built data extracts using bespoke SQL queries. These tools did not meet HIC's needs for managing data feeds of variable and changing data quality and structure, and producing reproducible extracts in a timely fashion. Therefore, in 2013, HIC examined available open source RDM tools (including testing i2b2 and tranSMART) to try to find a suitable alternative tool that provided a scalable solution for managing large volumes of heterogeneous data for multiple research projects, providing tools for curating and cleaning data as an integral part of the system, and utilising an integrated data management lifecycle. Existing RDM tools and off-the-shelf tools did not meet HIC's requirements for several reasons:

1. **Horizontal Scaling:** Most Extract Transform Load (ETL) tools are optimised for vertical scaling (more records) in a write-once per dataset solution in which transforms, data cleaning and optimisations are carried out once on each dataset hosted. HIC needed the ability to rapidly and incrementally curate many datasets at once, while also supporting rapid ETL and

extraction of heterogeneous one-off datasets such as those collected by the researchers themselves for specific projects (varying from patient-reported outcomes through complex clinical measurement to genomic and similar data).

2. **Data Cleaning and Curation Tools:** Many RDM tools are predicated on the external data sources being well-curated and the data being reliable and research-ready at the time of import to the analytics platform. The longitudinal datasets hosted by HIC have variable data quality and variable structure as underlying clinical systems change, and are subject to changing definitions over time as well as retrospective rewriting as individual patient's clinical diagnoses evolve. Therefore, the data requires significant restructuring and cleaning.
3. **Complex extraction transforms:** The requirement for extraction transforms can be implemented using existing technologies such as database views but these solutions lack scalability and curation.
4. **Data Lifecycle Management:** Most of the existing RDM systems have been implemented as a single data management resource, an instance of which could be accessed by many researchers. However, it was found that the cleaning and standardisation required by different groups depended on the research question and/or methods to be used and so a single data management resource fed from an all-purpose data cleaning and transformation processing pipeline would not meet the requirements.

The RDMP was therefore developed to address HIC's requirements. The integrated data management lifecycle separates out the RDMP's functionality into two related, but distinct activities: *Repository Data Lifecycle* and *Project Data Lifecycle* as illustrated in Figure 1. The *Repository Data Lifecycle* is involved with data preservation, metadata generation (feature extraction), data profiling and quality control, cohort discovery, data linkage and extraction. The *Project Data Lifecycle* is involved with data quality assessment and control, data transformation, and data analysis. In this design, the value chain is one in which the repository delivers value to the project through data extract and supply, and value is returned from the project through the capture and subsequent application of data transformation processes used in the project. The integrated data management lifecycle is applicable to all research data types rather than just clinical or biological data.

This paper describes the architectural features of the RDMP and evaluates both the impact of the implementation on HIC processes and the value of the system to the research community.

Data Description

High-Level Architecture

The RDMP is a systemic approach for the management of routinely collected healthcare and research data and the provision of cohort-specific extracts for research projects. The platform is a set of data structures and processes, sharing a core *Catalogue*, to manage electronic health records, genomic data and imaging data throughout their lifecycle from identification and acquisition to safe disposal or archival and retention in secured Safe Havens. The architecture components of the RDMP (shown in Figure 2 and described in Table 1) are a *Catalogue* and five internal processes (*Data Load, Catalogue Management, Data Quality, Data Summary*, and *Data Extraction*) that are designed to enforce rigorous information governance standards relevant to the processing and anonymisation of personal identifiable data. Only a summary of the processes is provided here with the details described in the online User Manual [14].

Table 1 - High-Level Architecture Components

**Catalogue**	The Catalogue contains a complete inventory of every dataset held in a given data repository including: a high-level description of each dataset; column level descriptions of data items; an inventory of validation rules, data transformations; export rules; outstanding dataset issues; supporting documentation; lookup information; and anonymisation rules. It utilises the *Load, Validation, Aggregate, Filter*, and *Transform* logics to drive all the five processes in the RDMP architecture.
**Data Load Process**	Establishes a single platform for data loading; manages remote data sources; loads data from structured and unstructured local sources; and includes reference data management for look-up based validation rules and condition-based searches as stored in the data catalogue. The process has a logging architecture that stores comprehensive data load details including row-level insert and update, archive locations, message-digest algorithm (i.e. MD5) of load files, user who loaded, any fatal errors, etc. The process also allows users to view which datasets have received loads, whether the load was successful or failed; and translates the structured *Load Logic* defined in the Catalogue into cleaning and anonymisation actions performed on data being loaded into the data repository.
**Catalogue Management Process**	This process is concerned with keeping the catalogue up-to-date, monitoring dataset issues and populating metadata for new datasets. The process is not unique to the Root DMN and it is intended that researchers keep their own copy of the catalogue up-to-date and provide feedback on new issues and transforms as they discover them. The catalogue management process captures and integrates useful contributions from researchers into the Root DMN Catalogue to further ensure that they are circulated amongst the entire research community.
**Data Quality Process**	This process is the core quality control function in the RDMP design. The process is focused on the development of data profiling and data quality assessment tools to monitor and report on the quality of the HIC-managed datasets, in terms of accessibility, access security, accuracy, completeness, consistency, relevancy, timeliness, and uniqueness.
**Data Summary Process**	This process creates summary layer aggregates for the data repository and data marts. The process creates discovery metadata through automated feature extraction and aggregation, generating what is essentially query optimisation metadata for the repository. It enables dataset discovery, dataset exploration, report generation, and cohort prospecting and generation.

**Data Extraction Process**	The data extraction process provides a structured means of versioning and releasing cohort-based datasets to researchers. In HIC's case, the release to researchers is often into a secure virtual "Safe Haven" environment where researchers can analyse the data and only export aggregate level results. However, providing data controllers allow it, the RDMP software is used to release data directly to researchers for analysis within other environments.<br>The data release process involves: auditing of data extraction (e.g. rows created, time started, any crash messages); retrieving and extracting of any global metadata documents specified in the Catalogue; sending dynamic SQL queries, created by the **Cohort Builder**, to the data repository; retrieving the result sets; creating an extraction time data quality report; extracting required lookup tables; and generating new catalogue entries tailored to the specified configuration in the Catalogue.

The Root Data Management Node (DMN) environment manages all of the data within the Data Repository. Subsets of data from the Data Repository are then provided for different research projects as data marts, along with a version of the Catalogue relevant to the data contained within the data mart. Data marts are project-specific or study-specific forms of a data warehouse [6, 8]. All of the processes (other than the Data Load Process) employed by the Root DMN environment can also be made available for each Branch Research Project DMN. The changes to the Catalogue made by a Research Project's DMN can be fed back into the Root DMN Catalogue and then be provided to other DMN Catalogues as required. Data are not shared between different data marts, but the logic of how to clean, transform and understand the data can be shared. This recognises that the value to be captured in the research process is in the metadata created by researchers to curate and extend the raw input of research data.

There are two export options provided by the software developed to support the data extraction process:

1. Researchers receive a branch RDM node complete with an empty Catalogue and a data mart. This is then populated with research data from the Root DMN data repository (transformed for anonymisation) and Catalogue information from the Root DMN Catalogue. All processes and accompanying software that runs on the Root DMN also works on researchers' project DMN instances. This allows researchers to perform extractions of their own (e.g. providing subsets of their research datasets to other researchers or students to perform additional analysis on the dataset, as shown in Figure 2).
2. Researchers receive their extracted datasets in flat file formats or as an extraction as an SQL database file. In this case, the descriptive metadata are provided to researchers in dynamically generated Word documents and Comma Separated Value (CSV) formatted lookup tables.

Privacy Handling

Ensuring identifiable data does not appear in data extractions is principally done by configuring which columns are extractable, which require special governance approval to be extracted and which contain patient identifiers (and therefore should not be extracted). This can be done once per dataset after which the rules will be applied to all project extractions. Since manual processes can be error prone the release pipeline also be adjusted to include further blanket checks. For example, adding the 'ColumnBlacklister' component to the default extraction pipeline allows specification of a Regular Expression which will block any data extractions containing columns matching the pattern (e.g. containing the word 'Id', 'Address' or 'Identifier'). It is also possible to write custom plugin data flow components. One such plugin component used by HIC looks for 10 digit sequences where the checksum matches the Scottish patient identifier CHI (Community Health Index) checksum algorithm in data being extracted. This prevents CHI numbers appearing in free text/unexpected fields from being extracted.

Data Access

The focus of this paper is the RDMP itself which can manage many forms of research data rather than the data managed by HIC's instantiation of the platform, but in brief, the datasets managed by the RDMP and hosted by HIC are generally sensitive clinical and research patient records and so are not openly available. Anonymised data extracts can be provided within a Safe Haven environment for specific cohorts to answer specific research questions given appropriate governance and ethical approvals. A list of the datasets currently hosted can be found at [13] and example datasets listed in Appendix A. To request access to the data please contact HIC [15].

Analyses

A data release is the process of linking relevant data for a specific cohort and providing an extract of data to researchers. Most projects require multiple data releases to update the same dataset as new data accrue, and/or to provide additional data as project needs change over time. HIC fully integrated RDMP into its existing work processes in July 2014 with regular updates and additional features being regularly added. To date (Dec 2017), 1321 data releases have been provided for research using the tool. There are currently 163 separate datasets which are loaded, managed and curated by the system. The largest data extract included data linked from 70 separate datasets.

Efficiency of Data Loading, Cleaning and Standardisation

Prior to the use of the RDMP, data loading, cleaning and standardisation effort was a time-consuming exercise due to the complexity of managing large numbers of continuously updating

datasets with varying structure over time. Data loading was highly manual and reactively undertaken in response to a researcher request for a linked dataset. The loading effort was often duplicated across projects. The RDMP, in contrast, provides a flexible pipeline to automate the process of loading data. The platform supports changing input formats e.g. when a dataset feed is supplied with a renamed column or no column headers. It also provides the framework for routine data cleaning.

To assess the impact on the efficiency of the data loading and management features of the RDMP, the mean number of hours spent on the task each year per data release were compared. Figure 3A shows that the total hours spent by the team on data loading, restructuring and management decreased significantly with the use of the RDMP tool, reducing from 24.6 hours per data release in 2013 to 3.0 hours in 2017. The tools for data cleaning were not in place in 2013 and so data was largely provided raw requiring duplicative cleaning by every research project analyst. With the use of the RDMP in 2014, a data cleaning project was undertaken with the effort reducing as data items were processed and cleaning automated. The overall effort for all supporting activities has reduced in line with the implementation of new features and improvements of RDMP: 5.6 hours of RDMP development and 3.0 hours for data management totalling 8.6 hours of supporting activity in 2017 per data release, versus 24.6 hours per data release in 2013 just spent on data management.

Number of Projects and Data Releases

Figure 3B shows that the total cumulative number of supported projects (where they have received one or more data release in any current or previous year, with recording starting from 2013) increased from 82 in 2013 to 533 in 2017. Between 2014 and 2017 approximately 140 (ranging from 139 to 146) unique projects were supported each year (where the project received at least one release that particular year). As many projects are multi-year the cumulative total number of projects supported is less than the addition of the number of projects supported per year.

Many projects require more than one data release in line with more data accruing over time and researchers carrying out longitudinal analysis. The number of new data releases since 2013 has increased each year. Release counts were captured for the last 8 months of 2013 and all months for 2014-2017. There were 142 data releases in 2013 (estimated to be 213 for the whole year) increasing to 456 in 2017. The cumulative number of total data releases assessed for this study increased from 141 in 2013 to 1647 by the end of 2017, of which 1321 were delivered using the RDMP.

Errors Rates and Types of Data Releases

Data releases were categorised into 5 types:

- First (planned): the first data release for a particular project
- Refresh (planned): refreshes of the data release with no changes except to include data that has newly accrued over time
- HIC Error: a data release to fix errors in a previous release caused by HIC making a mistake in interpreting the data specification
- Researcher Error: a data release to fix errors in a previous release caused by the research team making a mistake in the data specification
- Change Request: a data release including additional data fields requested by the research team after initial analysis of a data release which was correctly aligned to the data specification

The capability of the RDMP to improve the release of correct data was assessed by comparing the percentages of each type of release each year. Figure 3C shows the proportion of releases made to fix a HIC Error halved with the use of RDMP from 4.9% of releases in 2013 to 2.2% in 2017, because of improved reproducibility and error checking functionality within the RDMP. Similarly, the number of Researcher Errors reduced from 1.4% to 0.4%, and the number of change requests from 6.3% to 0.4%, both due to improved metadata and documentation prior to release supporting correct specification of the data required at first release. One of the features of the RDMP is the project and data specific documentation generated automatically on data extract. A word file is produced which provides all of the metadata for just the fields which have been extracted for the project along with project-specific summary charts and the logic used to build the cohort. The project-specific summary charts show gaps in the data that a researcher may not have been previously aware. Overall, the proportion of releases with correct data increased from 87.3% in 2013 to 96.9% in 2017.

Efficiency of Performing a Data Release

The RDMP Cohort Builder tool enables a Data Analyst to combine blocks/filters of best practice, standardised, re-useable SQL queries to build cohorts and extract data. The blocks can be reused by different Data Analysts for multiple projects rather than bespoke SQL code being written for every new project. The quantitative benefits of using the RDMP Cohort Builder and Data Extraction tool were measured by comparing the number of data releases produced each year and the time taken by Data Analysts to produce any release, and separately first and refresh data releases.

Figure 3D shows that the mean number of data releases per month increased steadily from 17.8 releases in 2013 to 38.0 in 2017. Figure 3D also shows that Data Analyst productivity significantly

increased, with a mean of 7.1 data releases carried out each month per FTE Data Analyst before RDMP implementation in 2013 compared to 25.3 in 2017. As the RDMP tools have improved over the years there has been an approximately three-fold increase in productivity levelling off over the last 2 years as the tool reaches as close to automation as possible with much of the remaining resource being the time taken working with researchers to document and define the required cohort.

Figure 4 shows that the hours spent on each data release varies widely. In 2013, over 75% of the data releases were completed with less than 5.9 hours of effort whereas in 2017 this has reduced to less than 2 hours. The mean time to produce a data release decreased from 5.7 hours in 2013 to 2.1 hours in 2017, with the median time decreasing from 2.5 hours to less than 1 hour over the same time period (Mann-Whitney U $p<0.001$). The maximum number of hours on a project decreased from 86.0 hours in 2013 to 49.9 in 2017. Figure 4 has been cropped at 45 hours excluding 3 releases, one in 2013 (86.0 hours), one in 2014 (98.2 hours) and the other in 2017 (49.9). The 2 earlier releases required complex cohort building with many iterative discussions with the researchers to define the cohort correctly. The release in 2017 was the first of a planned series of routine extractions of imaging data. This necessitated new development and many meetings with the imaging experts to ensure an accurate and easily repeatable extraction was created. The project with the second largest number of hours logged in 2017 took only 31 hours. There were 208 data releases with zero time marked against them. These were completely automated releases which took less than 5 minutes to initiate and so have no time booked against them. As might be expected, first releases took longer than refresh releases but there was a decrease in the average time to perform a data release for both types of releases.

Another reason for the reduction in time taken to produce data extract is the improved and standardised metadata held within the catalogue. The time spent with researchers in meetings to define the cohort has been reduced as researchers can now clearly see what data fields are available prior to being given their extract. This can help inform the criteria for the cohort and elucidate what fields are required in the data extract.

Number of Data Releases per Project

Figure 3D shows that there has been a steady increase in the average number of data extracts produced for each project due to the increased demand for new extracts, increasing from a mean of 1.7 data releases per project in 2013 to 3.1 in 2917. Figure 3C also shows that over 66.9% of the data releases in 2017 were refreshes of the data compared to 33.1% in 2013. Updating data from continuously accruing routinely-collected health data is particularly helpful for longitudinal studies

where maximising the length of follow-up is often a high priority. Prior to the development of the RDMP it was not feasible for HIC to provide regular refreshes in a timely fashion due to the manual effort to load new feeds and produce extracts for each project. Such studies could therefore only receive extracts every 1 to 2 years, which meant most studies were never refreshed as research funding is often shorter than this. The increased proportion of refreshed extracts was a result of reducing the waiting time for researchers and the improvements in the reproducibility of the data extract structure (see Qualitative Evaluation).

Qualitative Evaluation

There is a range of benefits of the RDMP which could not be measured quantitatively because either the metrics were not electronically recorded or because they were challenging to quantify. Therefore, the qualitative evaluation has been carried out by discussions with researchers and the team of Data Analysts.

Overall Efficiency

Prior to the implementation of the RDMP, there was a significant project backlog and it was estimated that it took approximately 6 months to provide a data release from when the research team requested the data, whereas as in 2017 this has reduced to several days (with approximately one less FTE working on the task). This was due to both changes in the efficiency of data loading and performing data releases (as quantitatively analysed above).

It used to take approximately 6 months to train a Data Analyst before they were able to independently load data and perform data releases. Using the RDMP this time has now reduced to a few weeks. The RDMP enabled the knowledge of the datasets and cohort building logic to be captured within the system metadata rather than just held by individuals. Junior Data Analysts can use the tool via a Graphical User Interface (GUI) rather than having to directly write SQL, with more senior Data Analysts developing and recording complex new filters, thus de-skilling the junior data analyst role.

Reproducibility

Prior to the RDMP, it was extremely challenging for Data Analysts using bespoke SQL scripts to provide the extracts in the same format each time especially when the data structures regularly changed at the source and a different analyst may have completed the subsequent work. Consequently, researchers needed to modify their analysis scripts to work with the new data structure each time a new extract was provided. This could take significant effort on the part of the

research team especially when trying to reproduce results. A core feature of the RDMP is the ability to provide data extracts in a reproducible structure over time.

A history of changes to data is stored. This information can be helpful to understand where data has been corrected/changed in the source system over time. Therefore, depending on researcher requirements a refresh data release which is required several years after the first release can provide the data with the values exactly as it was at the time of the first release or with the updated values in the 'live' source system (or both values if researchers need to compare them).

Data Quality Control

Overall data quality can be continually monitored, audited and improved by the Data Management team using the data quality tools within the RDMP. Data can be delivered to research projects with a confidence in quality that is testable and quantifiable. Although data profiling and monitoring are standard enterprise warehouse management techniques used in data control, quality monitoring, validity and anomaly identification, they are not activities that are well represented in the research data management life cycles. The data quality process provides the metrics that characterise and track stability and volatility in the research data. These metrics are then used to provide an automated assessment of the scope and conformance of the data to expectations before and after transformation processing.

Example Projects

The RDMP has been used to provide the data management and data extracts for a range of high impact recent publications such as [16-21].

Discussion

Over the last four years, the RDMP has been used to manage 163 clinical datasets (most of which are constantly accruing new data) and provided 1321 data releases for 420 different research projects. The RDMP has improved the provision of linked data extracts for research in several key ways:

- Researchers now receive metadata and documentation which is automatically generated and specific for the data fields they have received and/or requested. All processes are fully audited and documented along with data governance controls. The data quality of both the data repository and the research data extracts is testable and quantifiable using the RDMP tools.
- The mean time to produce a data release by Data Analysts decreased from 5.7 hours in 2013 to 2.1 hours in 2017. Data Analysts building cohorts and extracting data have become over 3

times more productive per FTE. The proportion of releases with correct data increased from 87.3% to 96.9%.

- The delivery time of a data extract from researcher data request has reduced from ~6 months to several days, primarily due to proactive and automated data loading, cleaning, curation and management. The RDMP has enabled highly complex projects to be delivered which were technically infeasible previously. The time required by researchers to clean and re-structure the data they receive has decreased as the data is delivered in the same structure at each new release which enhances reproducibility.

These improvements have not only benefited the research community but have also given additional comfort to the Data Controllers that their data is being robustly managed, as evidenced by positive feedback from regular Data Governance Committee Meetings with representation from Data Controllers. The controls, audit and logging functionality have provided supporting evidence contributing towards HIC attaining ISO27001 certification (an internationally recognised standard for information security management system) and to become a Scottish Government Accredited Safe Haven Environment.

We believe that the RDMP is unique in its clear separation of the *Repository Data Lifecycle* and *Project Data Lifecycle*. There are many other tools available which provide cohort building functionality or basic ETL functionality but they do not offer the same level of tight functional, and workflow integration the RDMP offers the data linkage community. One key benefit of the RDMP is the recognition that the data curation processes to identify, clean, correct, transform, and/or impute data in the datasets are integral in the RDM lifecycle and must be embedded in a highly structured and redistributable Catalogue so that the data cleaning can be performed on-demand and applied retrospectively to new cohorts.

Potential implications

The RDMP has been developed and utilised by a Scottish Safe Haven which handles both non-consented and consented linked datasets and provisions extracts for specific cohorts within a locked down researcher environment. The tools would be very helpful for other organisations who provide such a service. However, the tool could also be used by others who work in contexts with different data governance constraints and use different data. The tool is designed to manage continually accruing longitudinal data and so could be particularly helpful for groups who manage longitudinal cohorts. The RDMP could also be used for other data types than health data.

The RDMP is in active development. There are 2 other major additional work streams enabling the RDMP to handle Big Data: images and genomic data. The imaging plugin is currently in prototype and will be used to manage the Scottish National Radiology Dataset which includes over 23 million different examinations from a population of 5.4 million, with over 700 TB of data collected since 2006. The RDMP is currently being used to manage multiple "omic" data results from across Europe in a project to stratify patients with different types of Endocrine Hypertension and to manage the phenotypic data for widely used Bioresources such as GoDARTS [22]. Another area for development is further mapping data to international data standards such as Logical Observations Identifiers Names and Codes (LOINC), and Systematized Nomenclature of Medicine – Clinical Terms (SNOMED CT) [23]. This will help us to further restructure the laboratory datasets and improve semantic interoperability and the quality of cohort selection and data linkage [24]. We are also developing the researcher tools to work on the Research Project DMN for the management of diabetes datasets as part of an NIHR Global Health award which is establishing a major new Scotland-India clinical partnership to combat diabetes.

We are actively looking for other research groups with which to collaborate, especially, where the RDMP can be exposed to different types of data; data cleaning and transformation logic; metadata; data mapping and phenotype definitions. Collaborative projects have two main aims: (a) to assist each collaborator with their specific data management challenges; and (b) to improve the RDMP architecture by exposing the RDMP to different and diverse data requirements. We would welcome collaborations using the RDMP and any suggestions for new features.

Methods

The data for the evaluation method were obtained from HIC's customised JIRA issue and project tracking system [25] from May 2013 to the end of 2017. All the daily activities of HIC Data Analysts loading, cleaning and standardising data as well as preparing and releasing data extracts to researchers were recorded on timesheets within JIRA. The RDMP started to be used in production in July 2014 with regular updates and additional features being released every month.

The total number of projects is the number of unique research projects that have been supported. The time recorded by Data Analysts for a "data release" task includes all of the effort to discuss the requirements with research groups, document the requirements, produce code which defines the appropriate cohort, pull and link the relevant data for the cohort, anonymise the extract and to copy the data extract into the "Safe Haven" environment for researcher access.

All of the extract files obtained from querying the JIRA database are provided along with all of the statistical analysis in either excel or SPSS (SPSS, RRID:SCR_002865) in the supporting data and materials. A detailed description of how the results were calculated is also provided.

Availability of source code and requirements

- Project name: Research Data Management Platform
- Project home page: https://github.com/HicServices/RDMP
- Operating system(s): Windows
- Programming language: C#
- Other requirements: Microsoft SQL Server
- SciCrunch RRID (Research Resource Identification Initiative ID): Research Data Management Platform, RRID:SCR_016268
- License: GPL v3

User Documentation and Technical Details

The RDMP contains an extensive 95 page (20,658 words) User Manual. The RDMP ships with not only the dlls and pdb files required to debug it but also an embedded resource file containing all the source code of the RDMP.

Since all user interface classes are documented in the source code, the RDMP contains a feature which reads this documentation and screenshots each form resulting in a 166 page (30,864 words) Microsoft Word document (as of Nov 2017) with images and descriptions of all user interfaces in the application. Since these descriptions/images are created directly from the embedded source code at runtime they are never out of date and always reflect the version of software the user is using.

All messages and exceptions generated during runtime are recorded with a Stack Trace. This is combined with the embedded source code browser in the RDMP allows you to rapidly identify the source of problems in the program while it is running without needing a debugger.

The software suite for managing this database is written in C Sharp programming (i.e. C#) in a solution consisting of 63 projects and a codebase size of 96,000 lines of code supported by a unit testing harness with over 1070 tests. A core design philosophy of the Catalogue is to extend testability into all aspects of data curation. To this end, many modules support self-checking during runtime, thus allowing the user to quickly identify problems encountered during routine data activities.

Availability of supporting data and materials

The datasets supporting the results of this article are presented in a file named *Data and Analysis.rar* are available via the *GigaScience* GigaDB repository [26].

The RDMP User Manual is available publically at: https://github.com/HicServices/RDMP/wiki. The RDMP has its own test data generator which produces csv files suitable for testing and the user manual provides instructions for how to set this up.

Declarations

List of abbreviations

CSV: Comma Separated Value

Data Release: is the process where relevant data are linked for a specific cohort and an extract of data is provided for a research project

DMN: Data Management Node

ETL: Extract Transform Load

FTE: Full Time Equivalent

HIC: Health Informatics Centre

ISD: Information Services Division

JISC: Joint Information Systems Committee

RDM: Research Data Management

RDMP: Research Data Management Platform

SQL: Structured Query Language

Competing interests

The author(s) declare that they have no competing interests.

Funding

The authors acknowledge the support from the Farr Institute of Health Informatics Research and Dundee University Medical School. This work was supported by the Medical Research Council (MRC) grant number MR/M501633/1 (PI: Andrew Morris) and the Wellcome Trust grant number WT086113 through the Scottish Health Informatics Programme (SHIP) (PI: Andrew Morris). SHIP is a collaboration between the Universities of Aberdeen, Dundee, Edinburgh, Glasgow and St Andrews, and the Information Services Division of NHS Scotland. This project has also received funding from the European Union’s Horizon 2020 research and innovation programme under grant agreement No 633983 (PI: Maria-Christina Zennaro).

Authors' Contributions

EJ conceived and directed the RDMP project and drafted this manuscript. DS and TN designed the architecture. TN, CH, LT, GM, and PA designed and implemented the different modules of the architecture. MG produced all of the data from Jira for analysis, PR configured the tool for multi-omics data, WB populated the catalogue, implemented data standards within the catalogue and drafted some sections of this manuscript. JG acted as Product Champion providing requirements of the tool to support the Data Analysts, AD and BG provided clinical data expertise and many of the researcher requirements. All authors read, edited and approved the final manuscript.

Statement on Competing Interests

The authors have no competing interests to declare.

Appendix A: Sample inventory of HIC-managed datasets.

*** SMR = Scottish Morbidity Records**

Dataset	Description	Type of Data Stored
Accident and Emergency (A&E)	Accident and emergency data	Structured and non-coded data
Echocardiogram (ECHO)	Cardiology echocardiographic data	Structured and non-coded data
General Registry Office (GRO)	Official death certification data	Structured and coded data (i.e. ICD-9/10)
Laboratory	Laboratory data, comprising of biochemistry, haematology, immunology, microbiology and virology reports	Structured and coded data (i.e. Read Codes)
Master Community Health Index (CHI)	Demographic data including postcode of residence, General Practice registration, and date of birth/death	Structured and coded data (i.e. CHI Numbers, Postcodes and Health Boards)
Prescribing	All dispensed prescriptions for prescribed medications in primary care	Structured and coded data (i.e. British National Formulary (BNF))
Renal Register	Dialysis and transplant data	Structured and non-coded

		data
SMR00*	Scottish national hospital data for outpatients clinics	Structured and coded data (i.e. Specialty codes with occasional use of ICD-10)
SMR01*	Scottish national hospital data for inpatients clinics	Structured and coded data (i.e. ICD-9/10 and OPCS-3/4)
SMR02*	Scottish national hospital data for maternity admissions	Structured and non-coded data
SMR04*	Scottish national hospital data for psychiatric admissions and day cases	Structured and coded data (i.e. ICD-10)
SMR06*	Scottish national hospital data for cancer registration	Structured and coded data (i.e. ICD-10)
Stroke	All stroke admissions to the Ninewells Hospital Acute Stroke Unit	Structured and non-coded, but diagnoses are mapped to ICD-10
Vascular Laboratories	Duplex vascular ultrasound of Carotids and lower extremities	Structured and non-coded data

Appendix B

Case Scenario

The following case scenario describes, in practice, how the RDMP fits into the routine workflow of researchers and/or data analysts:

HIC receives a new dataset: 'blood sugar measurements' from the laboratory system. HIC populates the *Catalogue* with *Load Logic*, descriptive, structural and administrative data. As part of developing the load package, a HIC data analyst notices that one local laboratory system always supplies measures in millimoles per litre while the rest provide milligrams per 100 millilitres. The HIC data analyst creates an issue in the Catalogue under the new dataset and writes *Aggregate Logic* to highlight the problem in the records. After consulting with the data provider, HIC adjusts the Load Logic to standardise all measurements across the dataset, documents the adjustment in the Catalogue and marks the issue as resolved. As a final action, the HIC Data Analyst writes *Validation Logic* that will allow the *Data Quality Process* to identify any future measurements that are outside the expected range.

A short time later, a researcher, investigating a possible link between blood sugar levels and cause of hospitalisation, contacts HIC for cohort extraction. After obtaining the appropriate governance approvals, the *Data Extraction Process* is used to generate a new *Research Project DMN* consisting of a Catalogue and a *Data Mart* containing the extracted data and the accompanying tools to support research data management. The researcher uses the *Data Summary Process* to plot a number of key conditions and notices that there is an inconsistency in the coding of hospitalisation episodes over time. The researcher identifies that this has already been documented in the catalogue and decides that the best course of action is to map both codes to an international standard he/she is more familiar with. Through the *Catalogue Management Process*, the researcher defines *Transform Logic* that will map to the new scheme and documents the transform within the descriptive data in his/her catalogue. The researcher flags that this transform is of potential interest to other researchers. The new transform is reviewed and accepted by HIC and incorporated into the Transform Logic of the Root DMN as an option for any future extractions.

Figure Titles and Legends

Figure 1: Integrated Data Management Lifecycle

Figure 2: High-Level Architecture of RDMP

Figure 3: Comparisons of efficiency and errors from using the RDMP tool

Figure 3 Legend: A data release is a process where relevant data are linked for a specific cohort and an extract of data is provided for a research project. Figure 3A: Hours Spent on Different Activities Per Data Release. Figure 3B: Accumulative number of Projects, number of data releases for the period results were captured, normalised number of data releases estimated for whole years and the accumulative number of data release. Figure 3C: Proportion of Data Releases of Different Types. Data releases were categorised into First (first planned release for a new project), Refresh (planned release of the data release to an existing project with no changes but to include data that has newly accrued over time), HIC Error (release to fix errors in a previous release caused by HIC making a mistake in interpreting the data specification), Researcher Error (release to fix errors in a previous release caused by the research team making a mistake in the data specification) and Change Request (release including additional data fields requested by the research team after initial analysis of a data release which was correctly aligned to the data specification). Figure 3D: Mean number of data releases per month, mean number of data releases per month per FTE, and mean number of data releases per project.

Figure 4: Efficiency of performing data releases

Figure 4 Legend: The dark line in the middle of the boxes is the median. The bottom of the box indicates the 25th percentile. The top of the box represents the 75th percentile. The whiskers extend to 1.5 times the height of the box. The points are outliers, circles being outliers lying between 1.5 and 3 times the height of the box, asterisks being extreme outliers lying >3 times the height of the box.

Endnote References

1. Cox AM and Pinfield S. Research data management and libraries: Current activities and future priorities. Journal of Librarianship and Information Science. 2014;46 4:299-316.
2. Ball A: Review of the State of the Art of the Digital Curation of Research Data. http://opus.bath.ac.uk/19022 (2010). Accessed May 13, 2018.
3. Ball A: Review of data management lifecycle models. http://opus.bath.ac.uk/28587/1/redm1rep120110ab10.pdf (2012). Accessed May 13, 2018.
4. Corti L VdEV, Bishop L, et al. Managing and Sharing Research Data: A Guide to Good Practice. London, UK: Sage. 2014.
5. Poschen M, Finch J, Procter R, Goff M, McDerby M, Collins S, et al. Development of a Pilot Data Management Infrastructure for Biomedical Researchers at University of Manchester – Approach, Findings, Challenges and Outlook of the MaDAM Project. International Journal of Digital Curation. 2012;7 2:110-22.
6. Murphy SN, Weber G, Mendis M, Gainer V, Chueh HC, Churchill S, et al. Serving the enterprise and beyond with informatics for integrating biology and the bedside (i2b2). J Am Med Inform Assoc. 2010;17 2:124-30. doi:10.1136/jamia.2009.000893.
7. Lowe HJ, Ferris TA, Hernandez PM and Weber SC. STRIDE--An integrated standards-based translational research informatics platform. AMIA Annu Symp Proc. 2009;2009:391-5.
8. Brandt CA, Morse R, Matthews K, Sun K, Deshpande AM, Gadagkar R, et al. Metadata-driven creation of data marts from an EAV-modeled clinical research database. Int J Med Inform. 2002;65 3:225-41.
9. Li Z, Wen J, Zhang X, Wu C, Li Z and Liu L. ClinData Express--a metadata driven clinical research data management system for secondary use of clinical data. AMIA Annu Symp Proc. 2012;2012:552-7.
10. Harris PA, Taylor R, Thielke R, Payne J, Gonzalez N and Conde JG. Research electronic data capture (REDCap)--a metadata-driven methodology and workflow process for providing translational research informatics support. J Biomed Inform. 2009;42 2:377-81. doi:10.1016/j.jbi.2008.08.010.
11. Athey BD, Braxenthaler M, Haas M and Guo Y. tranSMART: An Open Source and Community-Driven Informatics and Data Sharing Platform for Clinical and Translational Research. AMIA Jt Summits Transl Sci Proc. 2013;2013:6-8.
12. Scheufele E, Aronzon D, Coopersmith R, McDuffie MT, Kapoor M, Uhrich CA, et al. tranSMART: An Open Source Knowledge Management and High Content Data Analytics Platform. AMIA Jt Summits Transl Sci Proc. 2014;2014:96-101.
13. Kimball P, Verbeke S, Flattery M, Rhodes C and Tolman D. Influenza vaccination does not promote cellular or humoral activation among heart transplant recipients. Transplantation. 2000;69 11:2449-51.
14. Nind T: RDMP User Manual. https://github.com/HicServices/RDMP/raw/master/Documentation/UserManual.docx (2017).
15. Kimball R, Ross, M., Thorthwaite, W., Becker, B., & Mundy, J. The data warehouse lifecycle toolkit.: John Wiley & Sons., 2008.
16. Fuchsberger C, Flannick J, Teslovich TM, Mahajan A, Agarwala V, Gaulton KJ, et al. The genetic architecture of type 2 diabetes. Nature. 2016;536 7614:41-7. doi:10.1038/nature18642.
17. Dreischulte T, Donnan P, Grant A, Hapca A, McCowan C and Guthrie B. Safer Prescribing--A Trial of Education, Informatics, and Financial Incentives. N Engl J Med. 2016;374 11:1053-64. doi:10.1056/NEJMsa1508955.

18. Myocardial Infarction G, Investigators CAEC, Stitziel NO, Stirrups KE, Masca NG, Erdmann J, et al. Coding Variation in ANGPTL4, LPL, and SVEP1 and the Risk of Coronary Disease. N Engl J Med. 2016;374 12:1134-44. doi:10.1056/NEJMoa1507652.

19. Gaulton KJ, Ferreira T, Lee Y, Raimondo A, Magi R, Reschen ME, et al. Genetic fine mapping and genomic annotation defines causal mechanisms at type 2 diabetes susceptibility loci. Nat Genet. 2015;47 12:1415-25. doi:10.1038/ng.3437.

20. The Scottish Government: A Charter for Safe Havens in Scotland. (2015). ISBN: 9781785444968

21. The Scottish Government. *Joined-up data for better decisions - Guiding Principles for Data Linkage*. 2012. ISBN: 9781782562153

22. Hébert HL, Shepherd B, Milburn K, Veluchamy A, Meng W, Carr F, et al. Cohort Profile: Genetics of Diabetes Audit and Research in Tayside Scotland (GoDARTS). International Journal of Epidemiology. 2017; doi:10.1093/ije/dyx140.

23. Bonney W, Galloway J, Hall C, Ghattas M, Tramma L, Nind T, et al. Mapping Local Codes to Read Codes. Stud Health Technol Inform. 2017;234:29-36.

24. Cox AM, Pinfield S and Smith J. Moving a brick building: UK libraries coping with research data management as a 'wicked' problem. Journal of Librarianship and Information Science. 2016;48 1:3-17.

25. Idso SB, Kimball BA, Pettit Iii GR, Garner LC, Pettit GR and Backhaus RA. Effects of atmospheric CO2 enrichment on the growth and development of Hymenocallis littoralis (Amaryllidaceae) and the concentrations of several antineoplastic and antiviral constituents of its bulbs. American journal of botany. 2000;87 6:769-73.

26. Nind, T; Galloway, J; McAllister, G; Scobbie, D; Bonney, W; Hall, C; Tramma, L; Reel, P; Groves, M; Appleby, P; Doney, A; Guthrie, B; Jefferson, E (2018): Supporting data for "The Research Data Management Platform (RDMP)" GigaScience Database. http://dx.doi.org/10.5524/100455

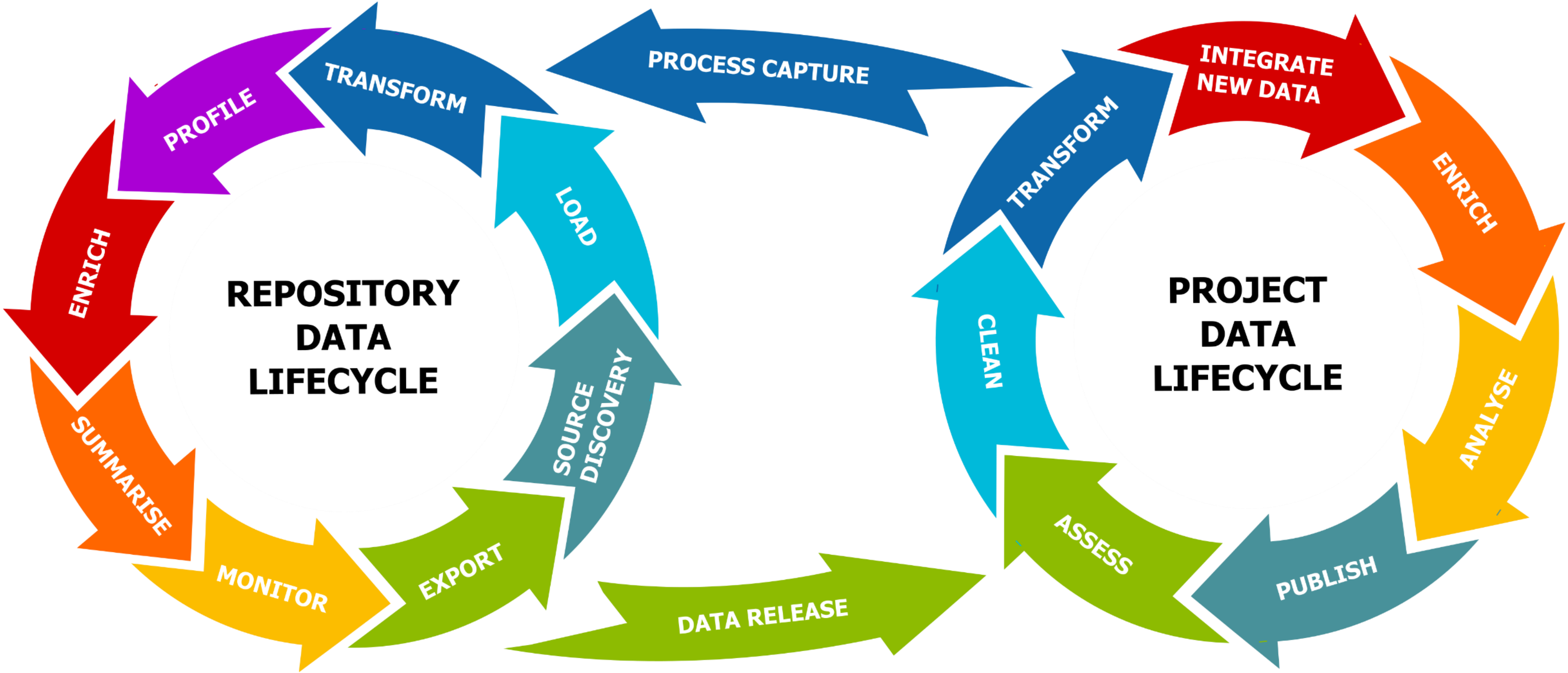
PROCESS CAPTURE
TRANSFORM
PROFILE
ENRICH
SUMMARISE
MONITOR
EXPORT
SOURCE DISCOVERY
LOAD
REPOSITORY DATA LIFECYCLE
DATA RELEASE
INTEGRATE NEW DATA
TRANSFORM
ENRICH
ANALYSE
PUBLISH
ASSESS
CLEAN
PROJECT DATA LIFECYCLE

Click here to downl

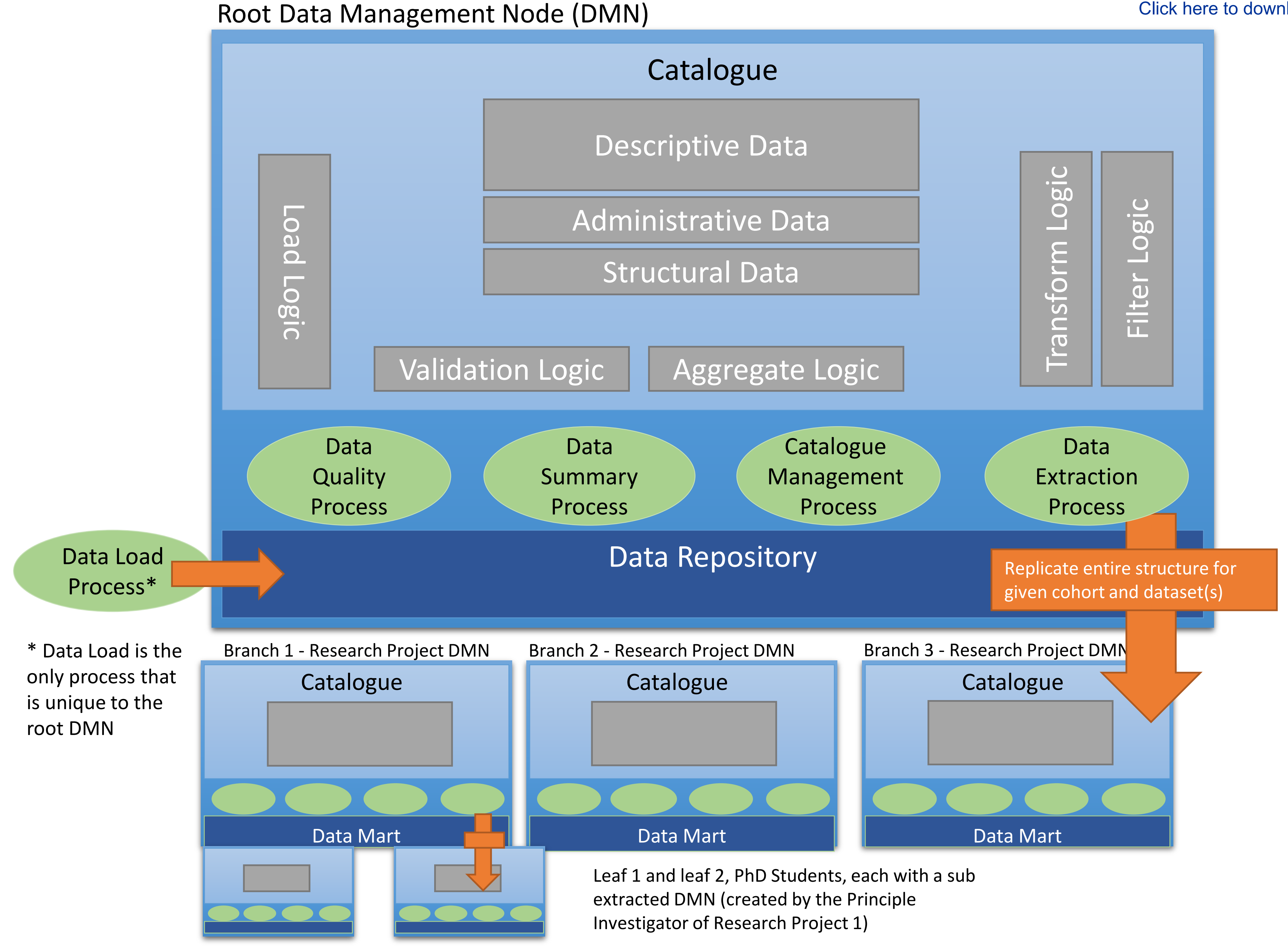

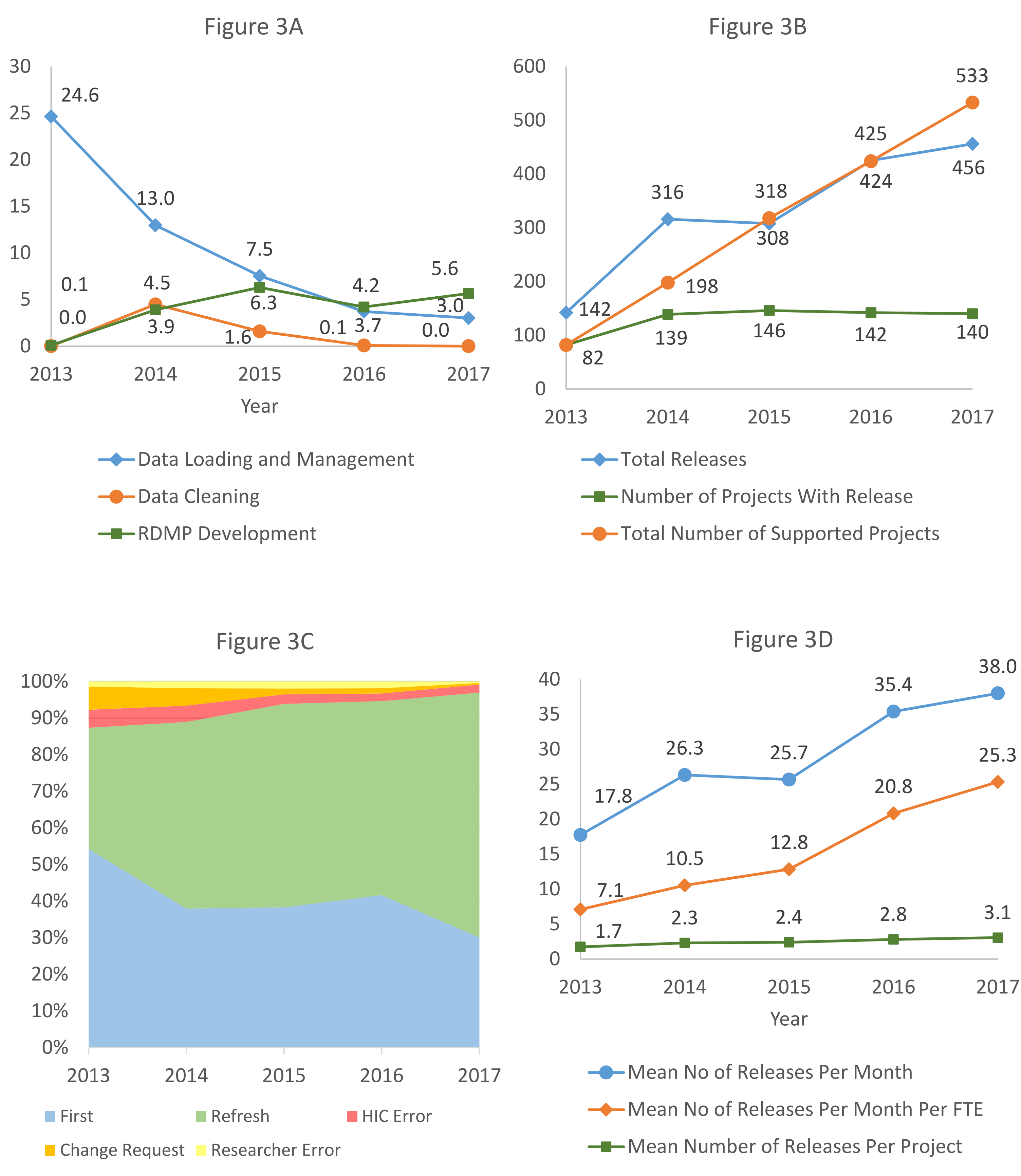

Figure 3A
24.6
13.0
7.5
0.1
4.5
4.2
5.6
0.0
6.3
3.0
3.9
1.6
0.1
3.7
0.0
2013
2014
2015
2016
2017
Year
Data Loading and Management
Data Cleaning
RDMP Development
Figure 3B
533
425
316
318
456
424
308
142
198
82
139
146
142
140
2013
2014
2015
2016
2017
Total Releases
Number of Projects With Release
Total Number of Supported Projects
Figure 3C
100%
90%
80%
70%
60%
50%
40%
30%
20%
10%
0%
2013
2014
2015
2016
2017
First
Refresh
HIC Error
Change Request
Researcher Error
Figure 3D
38.0
35.4
26.3
25.7
25.3
17.8
20.8
10.5
12.8
7.1
2.3
2.4
2.8
3.1
1.7
2013
2014
2015
2016
2017
Year
Mean No of Releases Per Month
Mean No of Releases Per Month Per FTE
Mean Number of Releases Per Project

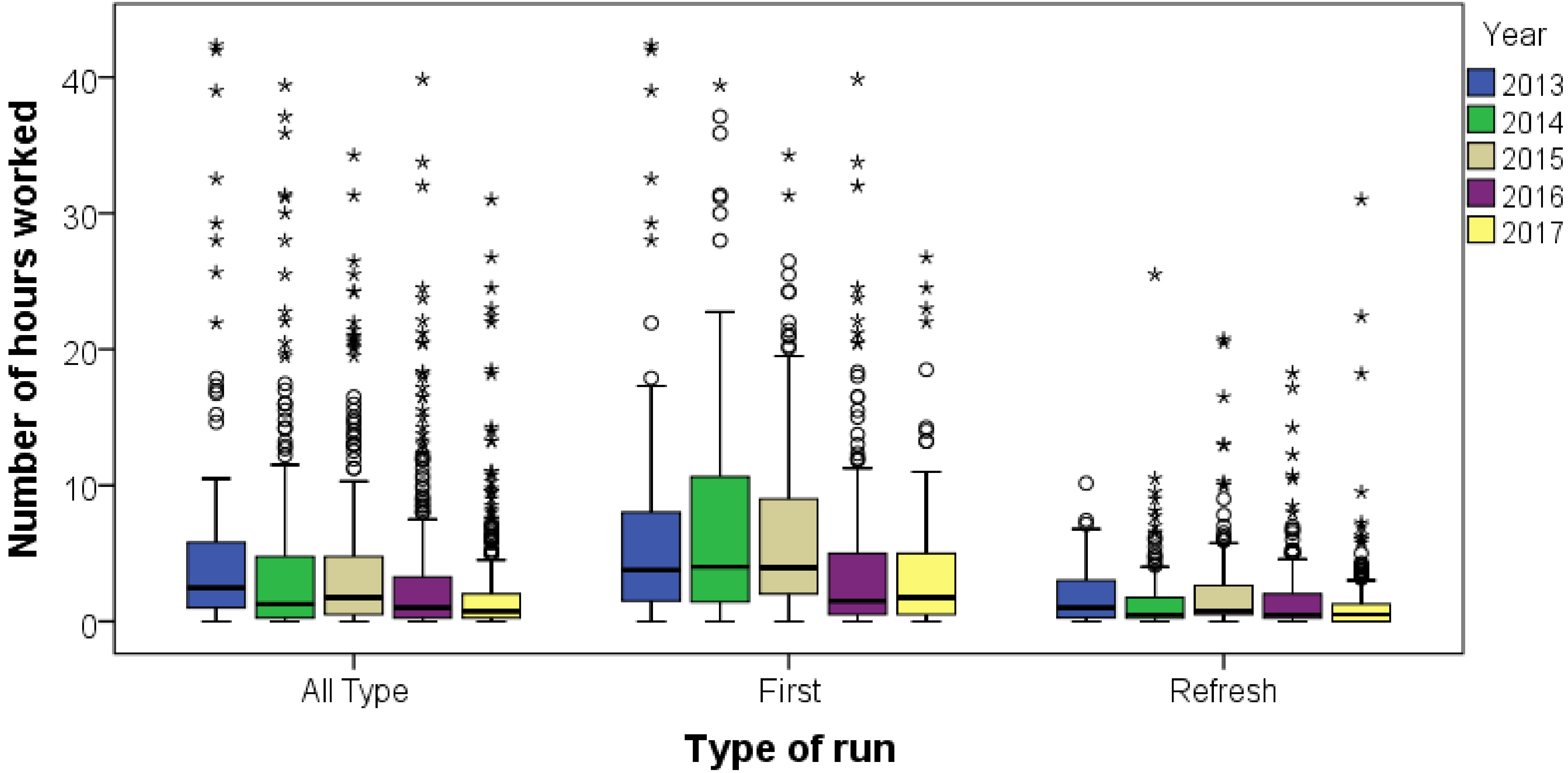

**Total Releases**	**142**	**316**	**308**	**425**	**456**	**77**	**111**	**118**	**177**	**136**	**47**	**161**	**171**	**225**	**305**
**Mean**	**5.7**	**4.2**	**4.0**	**2.9**	**2.1**	**8.2**	**8.6**	**7.0**	**4.3**	**4.2**	**2.1**	**1.6**	**2.2**	**1.7**	**1.2**
**Median**	**2.5**	**1.3**	**1.8**	**1.0**	**0.8**	**3.8**	**4.8**	**4.0**	**1.5**	**1.8**	**1.0**	**0.5**	**0.8**	**0.5**	**0.5**
**Max**	**86.0**	**98.2**	**34.3**	**39.9**	**49.9**	**86.0**	**98.2**	**34.3**	**39.9**	**49.9**	**10.2**	**25.5**	**20.8**	**18.3**	**31.0**
**75th Percentile**	**5.9**	**4.8**	**4.8**	**3.3**	**2.0**	**8.5**	**11.0**	**9.1**	**5.0**	**5.2**	**3.0**	**1.9**	**2.8**	**2.0**	**1.3**

Click here to access/download
Supplementary Material
Data and Analysis.rar
