# Supplementary material for: The research data management platform (RDMP): A novel, process driven, open-source tool for the management of longitudinal cohorts of clinical data
Source: Gigascience. 2018 May 22;7(7):giy060. doi: 10.1093/gigascience/giy060 (PMC6041881; doi:10.1093/gigascience/giy060)

# Method for Organising Data

out_multiRowsPerMonth_v5 contains the raw number of hours for each release.

The contents of out_multiRowsPerMonth_v5 were copied into new sheet called RawHoursByReleaseType in excel file “Summary Analysis”.

The analyses of the number of releases, sums of types of releases with all of the underpinning formula are shown in sheet AnalysisReleaseType.

The number of releases cumulating each year within projects are in raw file JIRADEV-111_2018-02-12_requestReleaseCountsByYear_cumulative. The number of projects with at least one release per year is shown in raw file JIRADEV-111_2018-02-12_requestWithOneOrMoreReleasesByYear. The data for these results were copied into tabs in excel file “Summary Analysis” and called RawReleasesPerProjectCumulative and RawProjectsWithOneOrMoreRelease respectively. The formulas for the analysis are shown in these tabs.

# How to make boxplot spss (v22)

Using file named RawHoursPerReleaseForBoxplot.xls

**Load file**

Define variables (Year, Hours, Release Type) – give hours and years names and populate the categories

(Year and Release type be marked as ordinal, leave hours as scale) (labels automatically given to years and Release Type but left empty for Hours)

**Graphs**


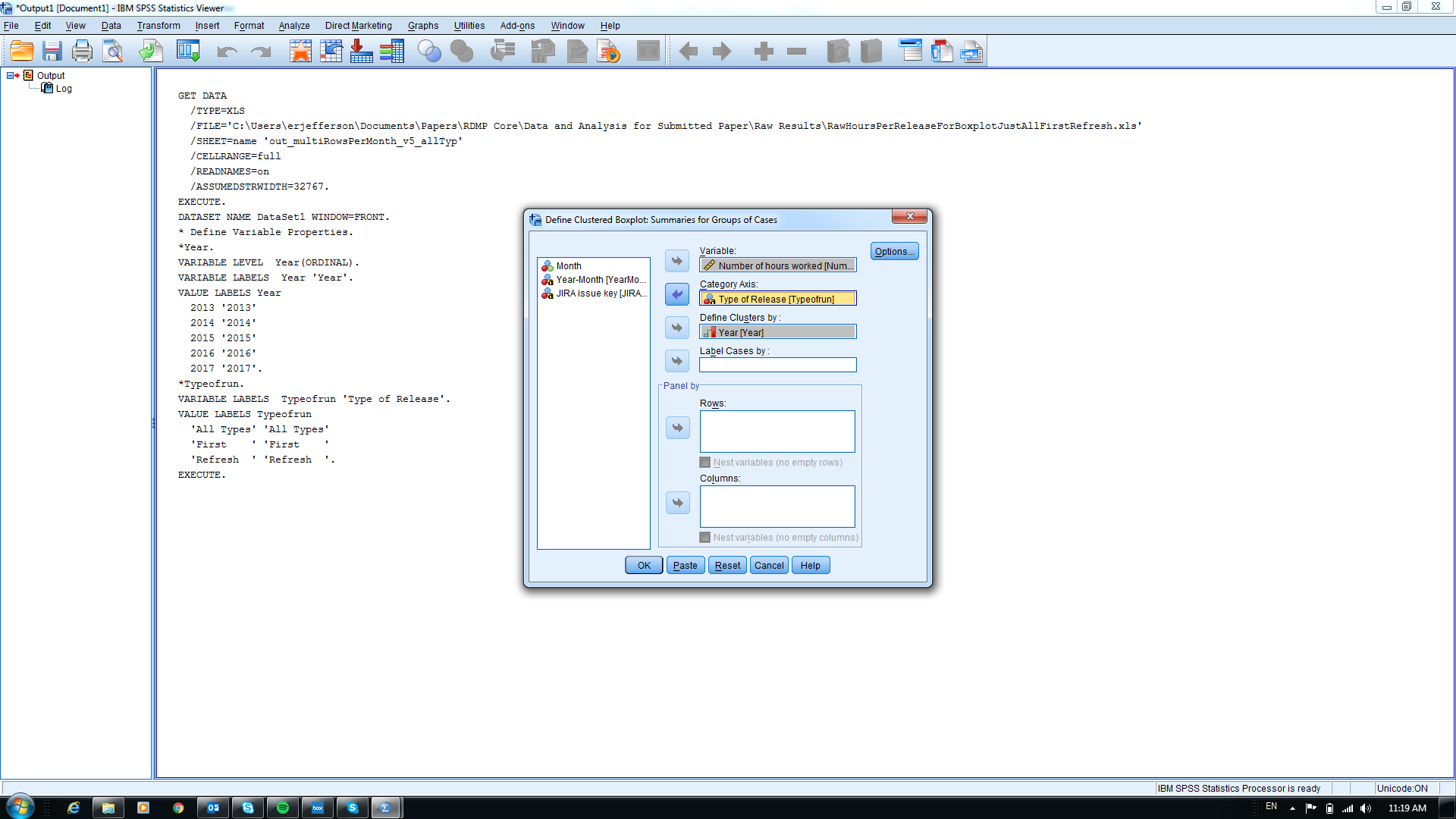


Graphs -> Chart Builder

Select boxplot (middle icon)

Hours on Y axis

Release type on X axis

Classify by Year (top right corner of graph)

Click OK to generate graph

Turn off numbers on points – Elements – unclick Show Data Labels

Double click on a bar – Change the variables so that Year is both colour and pattern then change bar options to increase the size of the bar

Double click on x-axis, go to categories and remove the Change, HIC error, External Error

Double click on background and change to white

Change the Y-axis to no decimal places


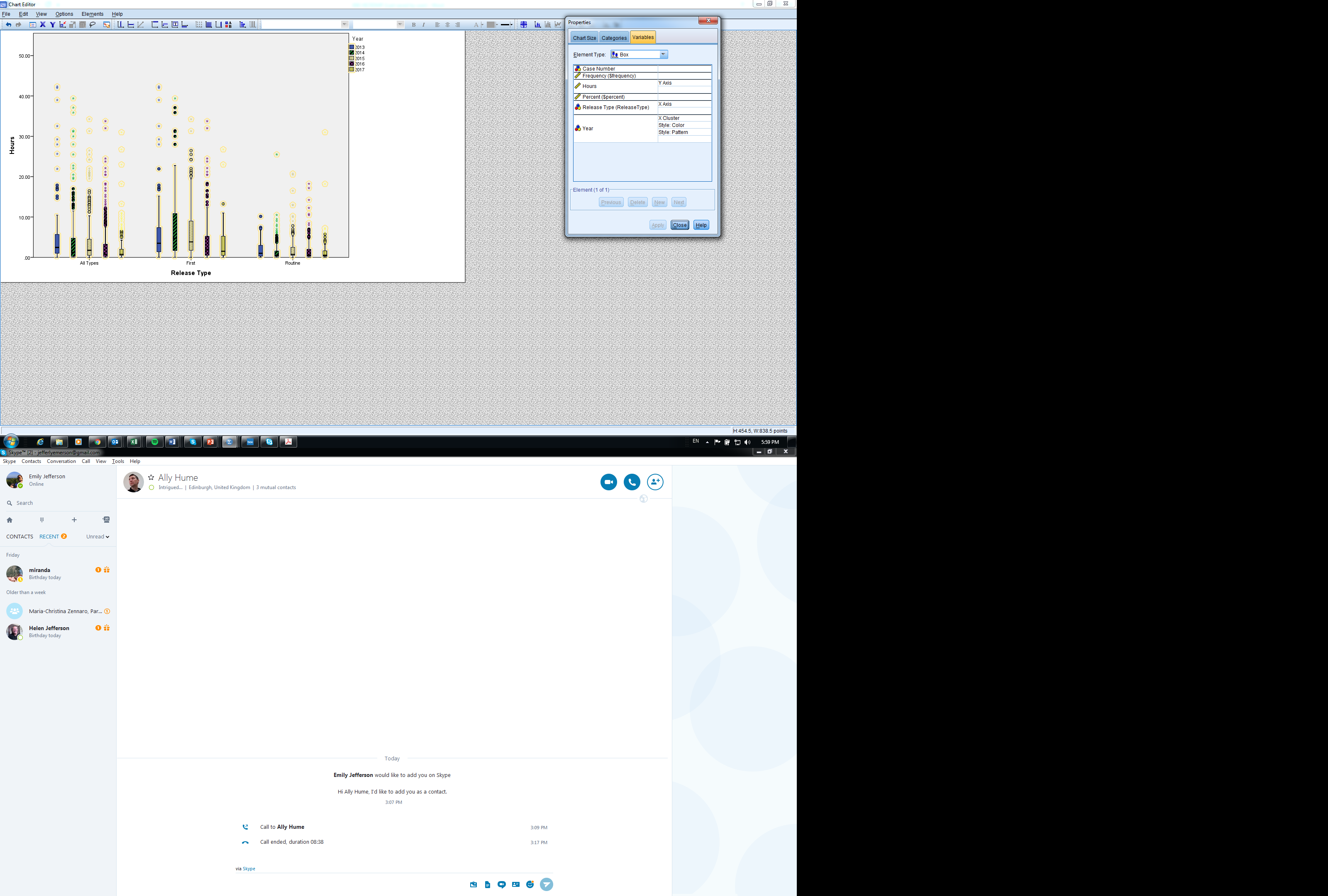


Didn’t actually need to remove the HIC Errors and other types from the graphs as could have used properties “Categories” and unticked some variables. Get to this by just double clicking on main chart.


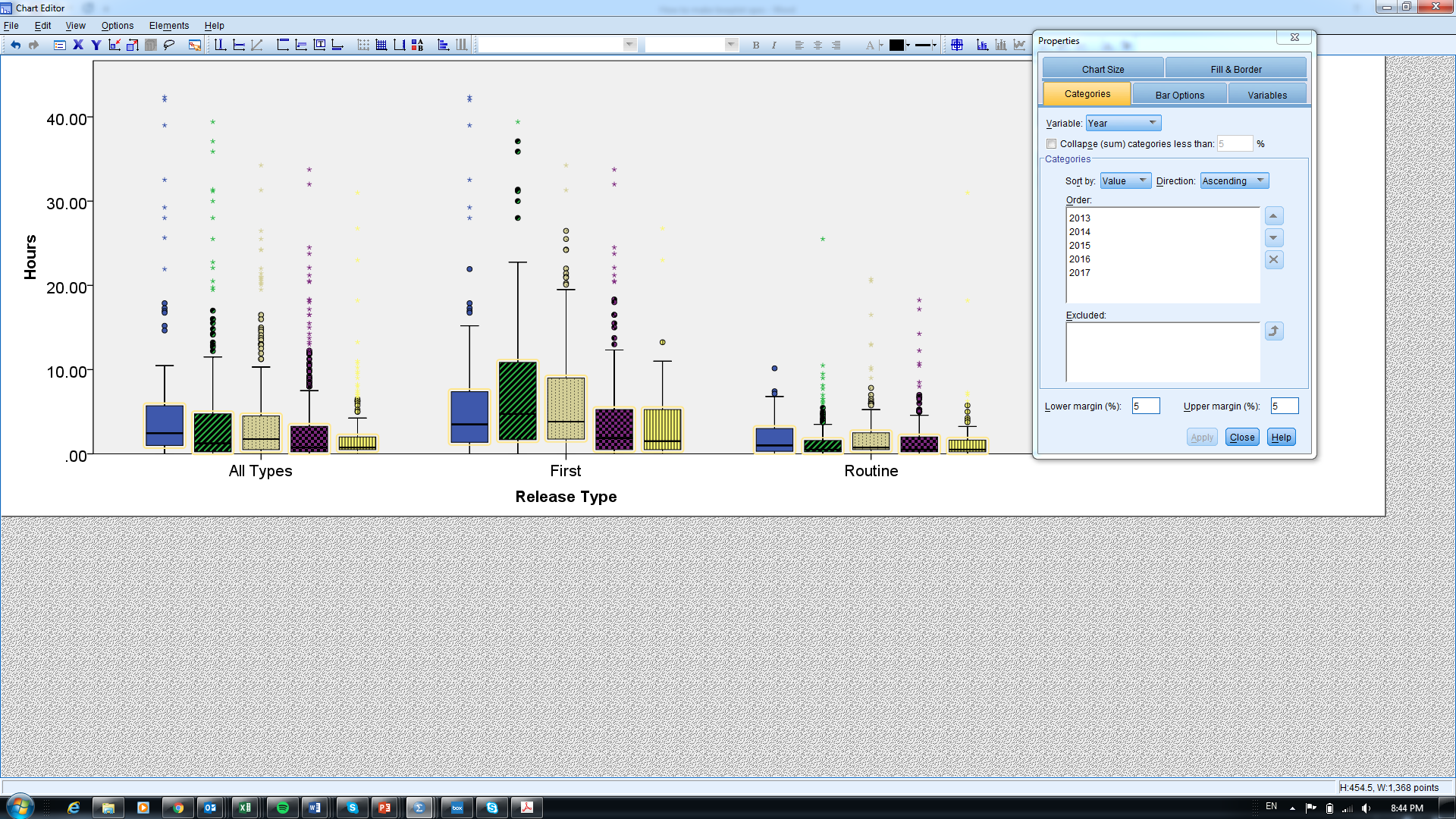


# Descriptive Analysis

A subset of the data in RawHoursPerReleaseForBoxplot was copied into 3 different files each with only “All Types”, “First” or “Refresh” results in: RawHoursPerReleaseForSummaryAllTypes.xls, RawHoursPerReleaseForSummaryFirst.xls, RawHoursPerReleaseForSummaryRefresh.xls. These results were then loaded into SPSS where Analyse -> Descriptive Statistics -> Explore was run with Year as Factor and Hours as Dependant list. Percentiles were selected from the Statistics Option. This was done for each type.


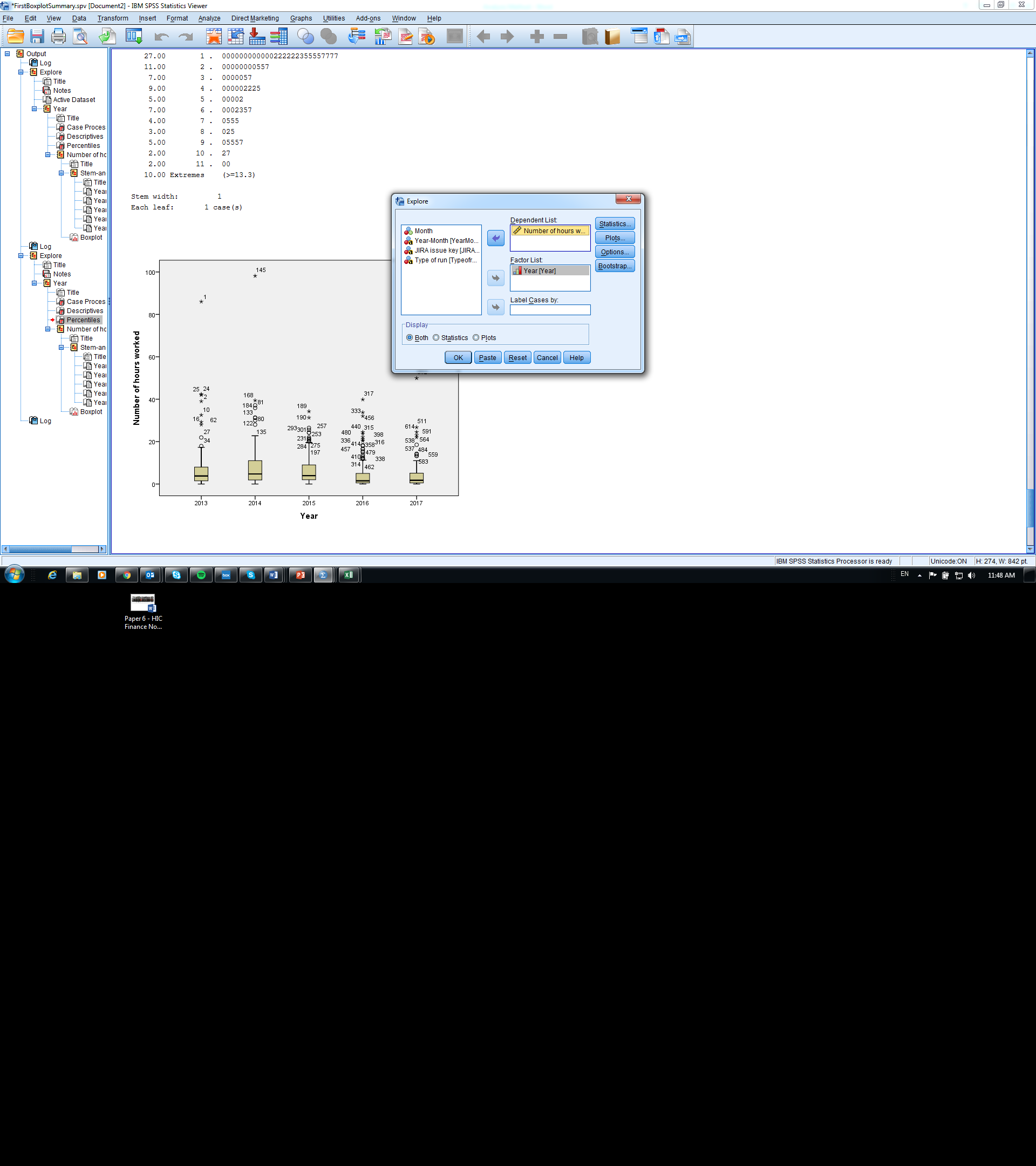


**Mann Whitney U Test**

Analyse -> Non Parametric Test -> Legacy Dialog -> 2 Independent Samples


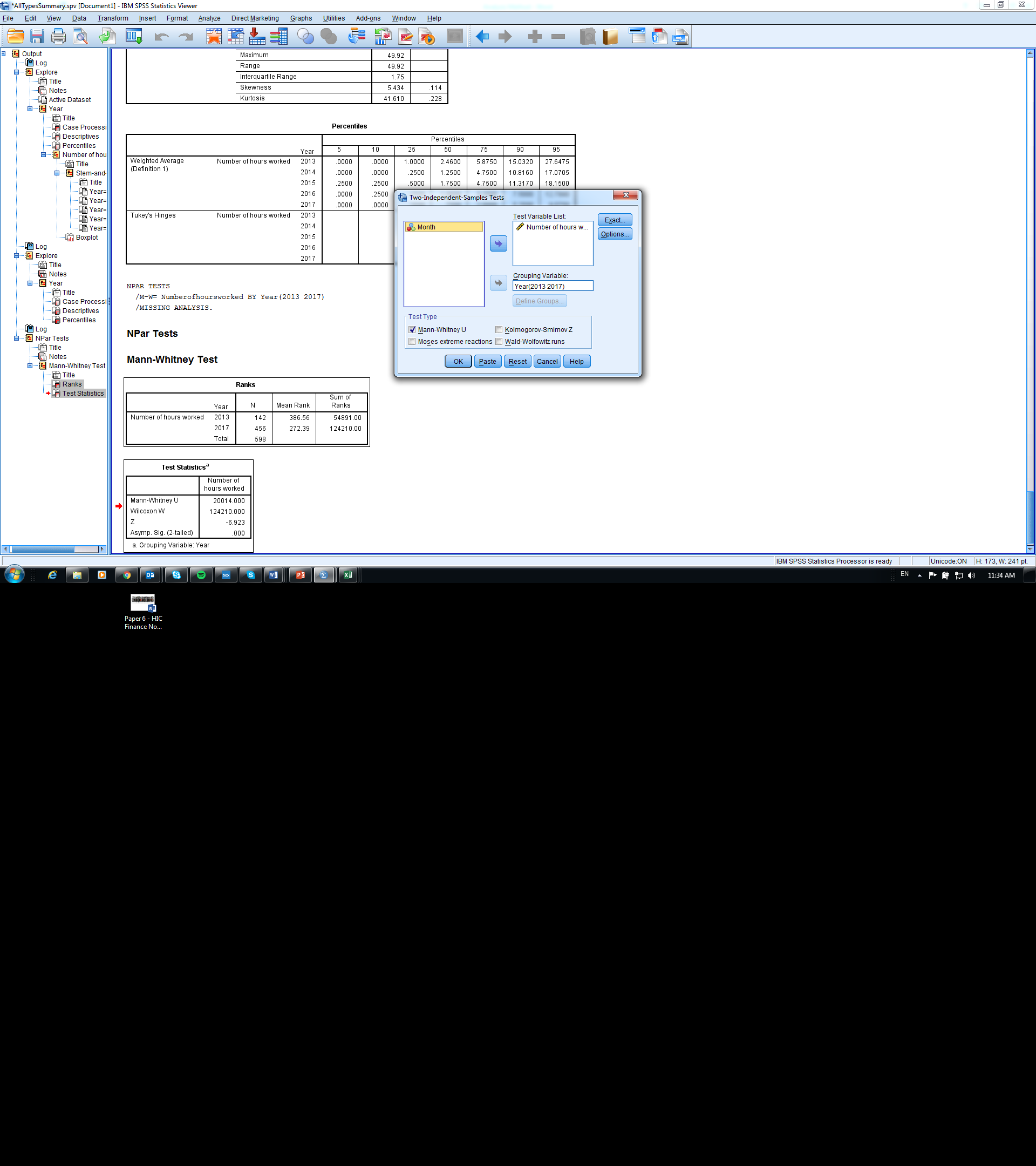

Supplement: Supplemental material [file giy060_supplemental_files.zip › Analysis Method.docx]
