# Supplementary material for: The research data management platform (RDMP): A novel, process driven, open-source tool for the management of longitudinal cohorts of clinical data
Source: Gigascience. 2018 May 22;7(7):giy060. doi: 10.1093/gigascience/giy060 (PMC6041881; doi:10.1093/gigascience/giy060)
Supplement: Response_to_Reviewer_Comments_Original_Submission.pdf [file giy060_response_to_reviewer_comments_original_submission.pdf]

Author's Response To Reviewer Comments

Close

Dear Dr Edmunds

Thank you for your email dated 4th May 2018 enclosing the reviewers' comments. We have carefully reviewed the comments and have revised the manuscript accordingly. Our responses are given in a point-by-point manner in the text below.

We hope the revised version is now suitable for publication and look forward to hearing from you in due course.

Sincerely
Emily Jefferson, PhD

1st Reviewer: No Changes suggested

2nd Reviewer

1) Length of background:
Reviewer comments: The background is quite long, and it takes a while to get to the "what". The problem should be well known to the journal audience, I don't need to be convinced that this is a significant challenge with importance to the larger research community. I would suggest getting to the description/solution (and figure 2) sooner.

Changes on p.3-4:
We have removed some superfluous words throughout the Background section.

We removed from the first paragraph:
"In the UK, there are a number of Research Data Management (RDMP) programmes sponsored by the Joint Information Systems Committee (JISC), a government-funded body charged with driving forward innovation and the use of digital technologies in UK higher education and research [6]. Although established in the humanities (Sudamih [7, 8]) and life sciences (ADMIRAL, [9]),"

We have removed from the section describing why the existing RDM tools and off-the-shelf tools did not meet HIC's requirements the following text:
•Data Cleaning and Curation Tools: "A considerable effort would be needed to develop a data cleaning and processing pipeline before being able to use many of the existing RDM tools."
•Complex extraction transforms: "If there are 10 optional transforms on a dataset which can be mixed and matched, then a view based approach does not scale to such a solution."
•Data Lifecycle Management: "during the requirements gathering stage of this project, the opinions of how to clean and standardise data were sought from many different researchers and clinicians who had previously been provided with research extracts for analysis by HIC. It was".

From the paragraph describing the Repository Data Lifecycle and Project Data Lifecycle we have removed: "Figure 1 summarises the integrated data management lifecycle used by the RDMP." and "Not all projects have to use the same transforms."

We have reduced the total length of the Background section by 17%.

2) How is privacy handled:
Reviewer Comments: I think it would be useful to add a section describing how privacy is handled. Will the ability to export a dataset without PII, a certifiable de-identified form or other option be added? Right now this seems to be the researcher's responsibility but it could be helpful to bake into the workflow and/or automate some elements of different jurisdictions etc. For example, could you recognize id's (SSN etc.), addresses, postcodes, phone numbers, email addresses and confirm they are meant to be included. Specify these elements and allow two forms of export (one de-identified and one identified), understandably this may be more suitable for a future release.

Changes on p.7.
We have added the following section:

"Privacy Handling
Ensuring identifiable data does not appear in data extractions is principally done by configuring which columns are extractable, which require special governance approval to be extracted and which contain patient identifiers (and therefore should not be extracted). This can be done once per dataset after which the rules will be applied to all project extractions. Since manual processes can be error prone the release pipeline also be adjusted to include further blanket checks. For example, adding the 'ColumnBlacklister' component to the default extraction pipeline allows specification of a Regular Expression which will block any data extractions containing columns matching the pattern (e.g. containing the word 'Id', 'Address' or 'Identifier'). It is also possible to write custom plugin data flow components. One such plugin component used by HIC looks for 10 digit sequences where the checksum matches the Scottish patient identifier CHI (Community Health Index) checksum algorithm in data being extracted. This prevents CHI numbers appearing in free text/unexpected fields from being extracted."

Recommended changes by Editor:
Please register any new software application in the SciCrunch.org database to receive a RRID (Research Resource Identification Initiative ID) number, and include this in your manuscript.

Changes made on page 15:
We have added under the Availability of source code and requirements section:
"SciCrunch RRID (Research Resource Identification Initiative ID): Research Data Management Platform, RRID:SCR_016268"

The instructions to authors states:
"GigaScience requires authors to deposit the data set(s) supporting the results reported in submitted manuscripts in a publicly-accessible data repository, such as GigaDB (see GigaDB database terms of use for complete details). This section should be included when supporting data are available and must include the name of the repository and the permanent identifier or accession number and persistent hyperlinks for the data sets (if appropriate)"

Changes made on page 15:
As per submission requirements we have uploaded the data used for the analysis of the paper
"The datasets supporting the results of this article are presented in a file named Data and Analysis.rar are available via the GigaScience repository: "The Research Data Management Platform (RDMP)" GigaScience Database. http://dx.doi.org/10.5524/100455."

Close
