# Supplementary material for: The research data management platform (RDMP): A novel, process driven, open-source tool for the management of longitudinal cohorts of clinical data
Source: Gigascience. 2018 May 22;7(7):giy060. doi: 10.1093/gigascience/giy060 (PMC6041881; doi:10.1093/gigascience/giy060)
Supplement: Reviewer_1_Report_(Original_Submission) -- Anne-Sophie Jannot — 4/20/2018 Reviewed [file giy060_reviewer_1_report_(original_submission).pdf]

Reviewer Report

Title: The Research Data Management Platform (RDMP): A novel, process driven, open-source tool for the management of longitudinal cohorts of clinical data

Version: Original Submission **Date:** 4/20/2018

Reviewer name: Anne-Sophie Jannot

Reviewer Comments to Author:

This paper describes the development and evaluation of the Research Data Management Platform (RDMP): an open source tool to load, manage, clean, and curate longitudinal healthcare data including high-dimension genetic data for research. This tool was conceived to better meet researcher expectation than currently existing platform, regarding management of longitudinal data and update of existing cohort. This platform enables to curate longitudinal and heterogeneous data with changing definition over time, to create metadata to keep trace for definition changes for instance, to perform several transforms of the data inside the RDMP. Other existing tools do not have to my knowledge all these capabilities. The authors state that implementation of this tool at the University of Dundee enables to considerably reduce time for data management and waiting time for researchers to receive data and that additional releases due to data misspecification drastically reduce. Documentation provided through github is highly comprehensive. This type of sharing of experience and feedback is very interesting for research institutions to be able to choose the better solution for research data management. The solution presented in this paper offers functionalities that are rarely available in other platforms. Several researches published in high impact journals used this platform to manage their data.The main limit of this paper is that authors lack feedback from other institutions and it is difficult to realize whether the provided material is self-sufficient to be able to use this platform in other institutions. But this paper is a first step to disseminate this platform which seems to provide a lot of functionalities meeting researcher expectations.

Level of Interest

Please indicate how interesting you found the manuscript: Choose an item.

Quality of Written English

Please indicate the quality of language in the manuscript: Choose an item.

Declaration of Competing Interests

Please complete a declaration of competing interests, considering the following questions:

- Have you in the past five years received reimbursements, fees, funding, or salary from an organisation that may in any way gain or lose financially from the publication of this manuscript, either now or in the future?

- Do you hold any stocks or shares in an organisation that may in any way gain or lose financially from the publication of this manuscript, either now or in the future?

- Do you hold or are you currently applying for any patents relating to the content of the manuscript?
- Have you received reimbursements, fees, funding, or salary from an organization that holds or has applied for patents relating to the content of the manuscript?
- Do you have any other financial competing interests?
- Do you have any non-financial competing interests in relation to this paper?

If you can answer no to all of the above, write 'I declare that I have no competing interests' below. If your reply is yes to any, please give details below.

No

I agree to the open peer review policy of the journal. I understand that my name will be included on my report to the authors and, if the manuscript is accepted for publication, my named report including any attachments I upload will be posted on the website along with the authors' responses. I agree for my report to be made available under an Open Access Creative Commons CC-BY license (http://creativecommons.org/licenses/by/4.0/). I understand that any comments which I do not wish to be included in my named report can be included as confidential comments to the editors, which will not be published.

I agree to the open peer review policy of the journal

To further support our reviewers, we have joined with Publons, where you can gain additional credit to further highlight your hard work (see: https://publons.com/journal/530/gigascience). On publication of this paper, your review will be automatically added to Publons, you can then choose whether or not to claim your Publons credit. I understand this statement.

Yes
