# Supplementary material for: The research data management platform (RDMP): A novel, process driven, open-source tool for the management of longitudinal cohorts of clinical data
Source: Gigascience. 2018 May 22;7(7):giy060. doi: 10.1093/gigascience/giy060 (PMC6041881; doi:10.1093/gigascience/giy060)
Supplement: Reviewer_2_Report_(Original_Submission) -- Brett Beaulieu-Jones — 5/3/2018 Reviewed [file giy060_reviewer_2_report_(original_submission).pdf]

Reviewer Report

Title: The Research Data Management Platform (RDMP): A novel, process driven, open-source tool for the management of longitudinal cohorts of clinical data

Version: Original Submission **Date:** 5/3/2018

Reviewer name: Brett Beaulieu-Jones

Reviewer Comments to Author:

This manuscript describes The Research Data Management Platform; an open source tool for managing clinical data sharing. It aims to solve the extremely important problem of cataloguing and storing clinical cohorts in a data warehouse amenable to ETL processes, data cleaning, tracking provenance, and sharing with permissioned clinical researchers. Of note, the data cleaning and ETL processes reported are more accurate and require significantly less time.The background is quite long, and it takes a while to get to the "what". The problem should be well known to the journal audience, I don't need to be convinced that this is a significant challenge with importance to the larger research community. I would suggest getting to the description/solution (and figure 2) sooner. I appreciate the release of source code and in particular it's organization, documentation and test generator. I think it would be useful to add a section describing how privacy is handled. Will the ability to export a dataset without PII, a certifiable de-identified form or other option be added? Right now this seems to be the researcher's responsibility but it could be helpful to bake into the workflow and/or automate some elements of different jurisdictions etc. For example, could you recognize id's (SSN etc.), addresses, postcodes, phone numbers, email addresses and confirm they are meant to be included. Specify these elements and allow two forms of export (one de-identified and one identified), understandably this may be more suitable for a future release.Overall, I found this manuscript and in particular the software project it describes to be well designed with a high potential to aid biomedical research.

Level of Interest

Please indicate how interesting you found the manuscript: Choose an item.

Quality of Written English

Please indicate the quality of language in the manuscript: Choose an item.

Declaration of Competing Interests

Please complete a declaration of competing interests, considering the following questions:

- Have you in the past five years received reimbursements, fees, funding, or salary from an organisation that may in any way gain or lose financially from the publication of this manuscript, either now or in the future?
- Do you hold any stocks or shares in an organisation that may in any way gain or lose financially from the publication of this manuscript, either now or in the future?

- Do you hold or are you currently applying for any patents relating to the content of the manuscript?
- Have you received reimbursements, fees, funding, or salary from an organization that holds or has applied for patents relating to the content of the manuscript?
- Do you have any other financial competing interests?
- Do you have any non-financial competing interests in relation to this paper?

If you can answer no to all of the above, write 'I declare that I have no competing interests' below. If your reply is yes to any, please give details below.

I declare that I have no competing interests.

I agree to the open peer review policy of the journal. I understand that my name will be included on my report to the authors and, if the manuscript is accepted for publication, my named report including any attachments I upload will be posted on the website along with the authors' responses. I agree for my report to be made available under an Open Access Creative Commons CC-BY license (http://creativecommons.org/licenses/by/4.0/). I understand that any comments which I do not wish to be included in my named report can be included as confidential comments to the editors, which will not be published.

I agree to the open peer review policy of the journal

To further support our reviewers, we have joined with Publons, where you can gain additional credit to further highlight your hard work (see: https://publons.com/journal/530/gigascience). On publication of this paper, your review will be automatically added to Publons, you can then choose whether or not to claim your Publons credit. I understand this statement.

Yes
